# Alterations in B Cell Compartment Correlate with Poor Neutralization Response and Disease Progression in HIV-1 Infected Children

**DOI:** 10.3389/fimmu.2017.01697

**Published:** 2017-12-01

**Authors:** Heena Aggarwal, Lubina Khan, Omkar Chaudhary, Sanjeev Kumar, Muzamil Ashraf Makhdoomi, Ravinder Singh, Kanika Sharma, Nitesh Mishra, Rakesh Lodha, Maddur Srinivas, Bimal Kumar Das, Sushil Kumar Kabra, Kalpana Luthra

**Affiliations:** ^1^Department of Biochemistry, All India Institute of Medical Sciences, New Delhi, India; ^2^Department of Pediatrics, All India Institute of Medical Sciences, New Delhi, India; ^3^Department of Pediatric Surgery, All India Institute of Medical Sciences, New Delhi, India; ^4^Department of Microbiology, All India Institute of Medical Sciences, New Delhi, India

**Keywords:** HIV-1, dendritic cells, B lymphocyte stimulator, B cells, long-term non-progressors, progressors, neutralizing activity

## Abstract

Several B cell defects are reported in HIV-1 infected individuals including variation in B cell subsets, polyclonal B cell activation and exhaustion, with broadly neutralizing antibodies elicited in less than 10–20% of the infected population. HIV-1 disease progression is faster in children than adults. B Lymphocyte Stimulator (BLyS), expressed on dendritic cells (DCs), is a key regulator of B cell homeostasis. Understanding how DCs influence B cell phenotype and functionality (viral neutralization), thereby HIV-1 disease outcome in infected children, is important to develop interventional strategies for restoration of B cell function. In this study, a total of 38 vertically transmitted HIV-1 infected antiretroviral therapy (ART) naïve children and 25 seronegative controls were recruited. Based on the CD4 counts and years post-infection, infected children were categorized as long-term non-progressors (LTNPs) (*n* = 20) and progressors (*n* = 18). Eight of these progressors were followed up at 6–12 months post-ART. Percentages (%) of DCs, B cell subsets, and expression of BLyS on DCs were analyzed by flow-cytometry. Plasma levels of B cell growth factors were measured by ELISA and viral neutralization activity was determined using TZM-bl assay. Lower (%) of myeloid DCs (mDCs), plasmacytoid DCs, and high expression of BLyS on mDCs were observed in HIV-1 infected progressors than seronegative controls. Progressors showed lower % of naive B cells, resting memory B cells and higher % of mature activated, tissue-like memory B cells as compared to seronegative controls. Higher plasma levels of IL-4, IL-6, IL-10, and IgA were observed in progressors vs. seronegative controls. Plasma levels of IgG were high in progressors and in LTNPs than seronegative controls, suggesting persistence of hypergammaglobulinemia at all stages of disease. High plasma levels of BLyS in progressors positively correlated with poor viral neutralizing activity. Interestingly on follow up, treatment naïve progressors, post-ART showed increase in resting memory B cells along with reduction in plasma BLyS levels that correlated with improvement in viral neutralization. This is the first study to demonstrate that reduction in plasma BLyS levels correlates with restoration of B cell function, in terms of viral neutralization in HIV-1-infected children.

## Introduction

Slower disease progression or suppression of viremia in HIV-1 infected individuals is linked with “protective” host factors like the 32 base pair deletion of the co-receptor CCR5, and the presence of specific human leukocyte antigen (HLA) class I alleles (HLA-B57 and HLA-B27) (1–3). However, progression rates are not much influenced by HLA class I variation in pediatric infection, wherein AIDS typically develops faster than in adults (2, 4–6). Infected children have persistent high viremia, delayed viral set point, presumably in part due to an immature immune system, and the rapid expansion of CD4^+^ T cells (3, 7, 8). Also, in early stages of life, lack of immunological memory and tolerogenic state of innate and adaptive immunity, render the host more susceptible to infectious pathogens like HIV-1 (7). Despite this, some infected children (<1%) maintain stable CD4 counts and remain asymptomatic for more than 7 years of infection [long-term non-progressors (LTNPs)] and their immune activation is low (8–10). Innate immunity is the earliest defense mechanism preventing microbial infection and alterations in which may impact mother to child transmission and disease outcome (11). The pediatric model affords several advantages for studying virus-host interactions, including the information on virus source and time of exposure (11). In infants who are exposed to HIV-1, innate immunity is of particular relevance in conferring protection since the adaptive immune system is still under development (12, 13). There is a paucity of information on the interactions between innate and adaptive immune response in HIV-1 infection that influences disease progression in infected children.

Dendritic cells (DCs) are one of the earliest cell types that encounter HIV-1 at mucosal sites (14). Given that DCs are professional antigen-presenting cells and form a critical link between the innate and the adaptive immune responses, it is likely that they possess an inherent capacity to modulate the balance between tolerance and protection (15). The two major DC populations in peripheral blood are CD11c^+^ myeloid DCs (mDCs) and CD123^+^ plasmacytoid DCs (pDCs) (16–18). Further, subsets of mDCs, i.e., BDCA-3^+^ DCs and BDCA-1^+^ DCs have been described (19, 20). In HIV-1-infected individuals, a reduction in circulating mDC subsets and pDCs has been shown earlier (19, 21). Decrease in mDC subsets has also been observed during advanced SIV infection (22). The overall outcome of HIV-1 infection may depend on the ability of the host to maintain DC homeostasis at mucosal sites. A number of B cell alterations are seen in HIV-1 infected individuals like polyclonal activation, exhaustion or loss of memory B cells, and a global impairment of antibody (Ab) responses (23–25). These B cell perturbations lead to functional abnormalities, as demonstrated by hypergammaglobulinemia, decreased B cell responsiveness to both T cell-dependent and T cell independent antigens and compromised responses to vaccination. The specific mechanisms contributing to B cell abnormalities are only partially known, and multiple factors may account for their dysfunction (25–27). HIV-1 driven alteration of the cytokine and chemokine milieu has been described as a predisposing factor of B cell dysfunction (28). In a recent study by Mabuka et al. (29) conducted in 22 women from the KwaZulu-Natal province of South Africa, pre- and post-HIV-1 subtype C infection, B cell defects were seen in hyperacute HIV-1 infection in antiretroviral naïve status, that are abrogated with immediate treatment, implicating the influence of viremia on B cell alterations. However, higher levels of CXCL13 and not B cell changes or viremia during hyperacute infection were found to be associated with emergence of cross-neutralizing antibodies within 1 year of infection (29).

The DCs influence mucosal B cell responses against HIV-1 through contact and/or production of B cell growth factors such as B Lymphocyte Stimulator/B cell-activating factor belonging to the TNF family (BLyS/BAFF), which in turn may lead to skewed B-cell subsets and poor outcome of B-cell responses against the virus, thereby modulating threshold toward disease progression (30, 31). Study on HIV-1/SIV infections in non-pathogenic animal models and from mucosal challenges in non-human primates suggests the importance of maintaining mucosal immunity in conferring protection (32).

Although there is sufficient B cell functionality to mount broadly neutralizing antibodies (bnAbs) against HIV-1, only a small proportion of HIV-1 infected individuals elicit bnAb responses (33). HIV-1 specific bnAbs play a crucial role in viral neutralization and mediate effector functions such as Ab-dependent cell-mediated cytotoxicity (ADCC) and thereby augment antiviral responses (34). Viral determinants are not the only factors driving infant neutralizing antibody (nAb) breadth, rather host determinants are equally important. Defining the immune correlates that influence the generation of bnAbs will contribute to the design of efficient immunotherapies (10).

There is limited information on phenotypic and functional defects of DCs, B cells and DC mediated alterations in B cell subsets in pediatric HIV-1 infection and disease progression (15, 17, 25, 27, 28, 35, 36). Independent studies have reported lower percentages of DCs and linked DC-mediated alterations of B cell subsets with faster disease progression (32), while preservation of HIV-1 specific memory B cells is associated with efficient viral neutralization in elite controllers (ECs) (37). Delineating the innate mechanisms that contribute to improvement of B cell function in HIV-1 infected children can provide new leads into vaccine development.

This is a comprehensive study undertaken to evaluate alterations in innate and humoral immune responses by profiling DCs and B cell subsets and their modulatory effect on viral neutralizing antibody response in chronic HIV-1C infected children at different stages of disease progression.

## Materials and Methods

### Study Subjects

A total of 38 HIV-1-infected children and 25 seronegative controls were recruited in this study. After obtaining written informed consent from parents/legally accepted representative (LAR), blood samples (2–4 mL) of HIV-1 infected children and seronegative donors were collected in ethylenediaminetetraacetic acid (EDTA) vacutainers from the Pediatrics out Patient Department (OPD) and Pediatrics Surgery OPD, respectively, at the All India Institute of Medical Sciences (AIIMS), New Delhi. The HIV-1 infected ART naïve children were grouped into 20 LTNPs and 18 progressors of which 8 progressors were followed up post 6–12 months of initiation of ART. HIV-1 infected asymptomatic ART naïve children with >7 years of infection, with CD4 counts ≥450 cells/μL at the time of recruitment, and stable for the last 18 months, were categorized as LTNPs. Progressors were defined as infected children (age <5 years) with CD4 count ≤25% and children (age >5 years) with CD4 counts ≤450 cells/μL or declining since 18 months prior to recruitment (10, 38, 39). The ART regimen for the infected children on treatment included a combination of two nucleoside reverse transcriptase inhibitors (NRTIs)—Lamivudine with Zidovudine or Stavudine and one non-nucleoside reverse transcriptase inhibitor (NNRTI)—Nevirapine or Efavirenz, as per the national guidelines.

### CD4 Count and Viral Load Measurement

HIV-1 viral loads were determined in plasma samples of infected children by quantitative reverse transcriptase polymerase chain reaction (Quantitative RT-PCR) (Roche COBAS TaqMan HIV-1 v2.0; Roche Diagnostics). The lower detection limit of the assay was 47 HIV-1 RNA copies/mL. The CD4^+^ T cell counts were assessed by flow-cytometric analysis (BD Biosciences) at the Department of Microbiology, AIIMS.

### Measurement of Plasma Levels of IgG, IgA, BLyS, IL-4, IL-6, IL-10

Plasma levels of IL-4, IL-6, IL-10 and BLyS were measured with a commercially available ELISA kit (R&D Systems) according/to the manufacturers’ protocol. Plasma levels of IgA were measured using a commercially available kit from Immunology Consultants Laboratory, Inc. Plasma levels of IgG were determined using ELISA kit (Raybiotech) according to the manufacturer’s protocol.

### Flow-Cytometric Analysis of DC Subsets, Membrane Expression of BLyS, and B Cell Subsets by Surface Staining of DC and B Cell Subsets

**Figure 1:**
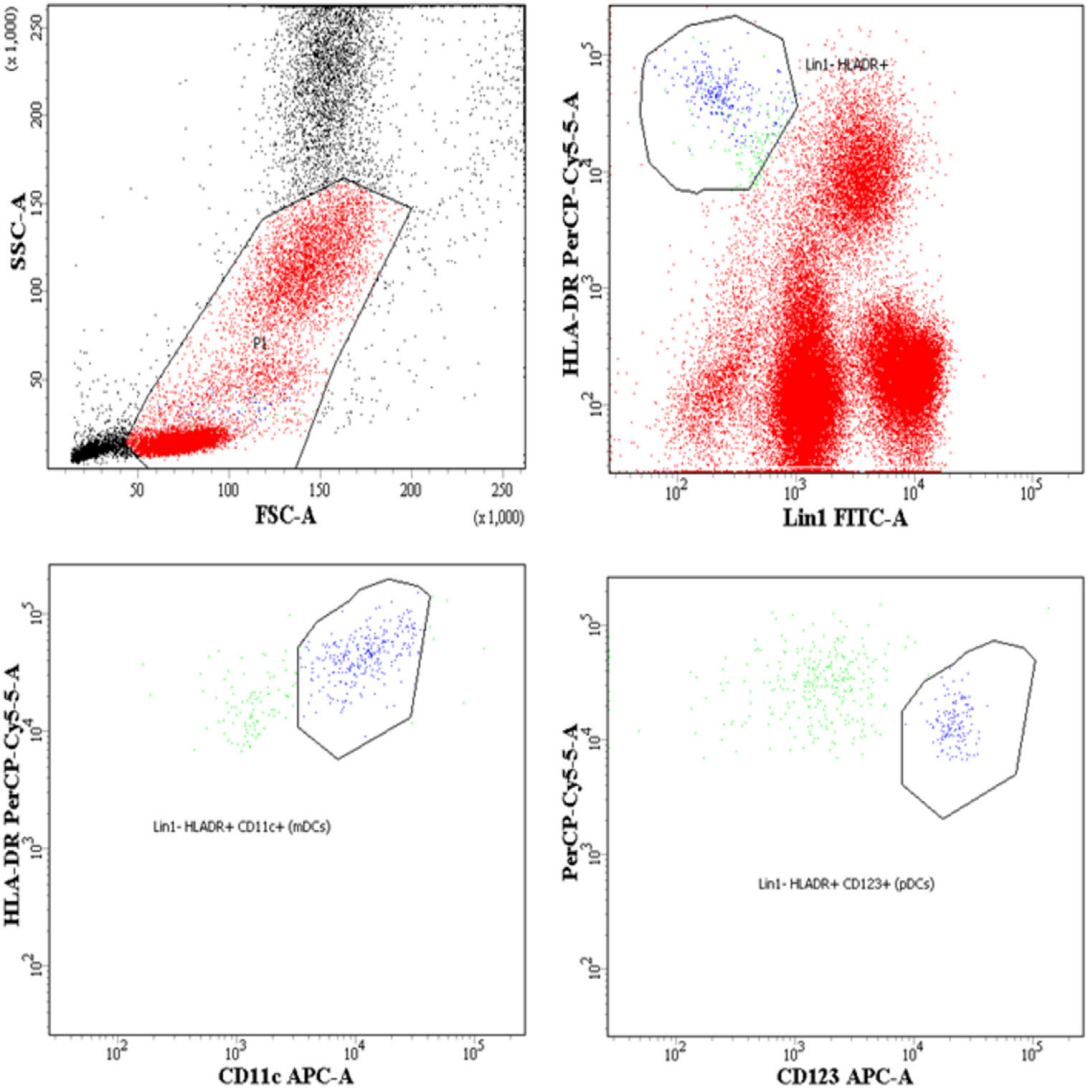
Flow-cytometric analysis of dendritic cell (DC) subsets in whole blood and surface expression of B lymphocyte stimulator (BLyS) on myeloid DCs (mDCs) and plasmacytoid DCs (pDCs). Representative gating strategy for DC subsets. Cells were gated on whole blood, mDCs were defined as Lin1 (CD3, CD14, CD16, CD19, CD20, and CD56)^−^ HLADR^+^ CD11c^+^, pDCs were defined as Lin1^−^ HLADR^+^ CD123^+^. Percentages of mDCs and pDCs are expressed as % of whole blood (leukocytes), which includes lymphocytes, monocytes, and granulocytes.

Whole blood (100 μL) was subjected to surface staining by adding the following Ab cocktails: Lineage-1 (cocktail of CD3, CD14, CD16, CD19, CD20, and CD56) FITC, CD11c/CD123 APC, HLA-DR PerCPCy5.5, BLyS PE. The Lineage Cocktail 1 used in FCM analysis contained Ab clones that, in combination, stained lymphocytes, monocytes, eosinophils, and neutrophils. Cells that were negative for staining with Lin1 and positive for HLA-DR, CD 11c/CD123 were identified as DCs. For B cell phenotyping, cocktail of antibodies used was CD19 FITC, CD21 PE, CD27 APC and CD10 PerCPCy5.5. Isotype controls used for antihuman CD11c, CD123, and CD10 antibodies are shown in supplementary data (Figure S1 in Supplementary Material). The panel of antibodies used to identify the subsets of DCs and B cells is detailed in Table S1 in Supplementary Material. Tubes were vortexed and incubated at room temperature for 30 min. After incubation, red cells were lysed using BD lysing solution followed by washing with 2 ml of 1X phosphate buffered saline. Cell pellets were resuspended in 300 µl 4% paraformaldehye and stored in dark at 4°C, prior to flow-cytometric analysis. Acquisition was performed on FACS-CANTO using FACSDIVA as software. A total of 100,000 events were acquired for each sample during FCM analysis. Figure 1 shows representative gating strategy of DC subsets, Figure 3 shows representative gating strategy of B cell subsets.

### Viral Neutralization Assay

Plasma samples from 28 antiretroviral naïve (10 LTNPs, 18 Progressors) HIV-1 infected children at baseline, and 8 follow-up samples were assessed for cross neutralization activity in a single-round HIV-1 envelope pseudovirus (200 TCID_50_) infection of TZM-bl cells as described elsewhere (40). Viruses (*n* = 11) of tier 1 and 2 belonging to different clades (41) (Table S2 in Supplementary Material) were used to measure viral neutralizing activity. HIV-1 envelope pseudoviruses were produced by co-transfecting HEK293T cells with HIV-1 envelope containing expression vector and an HIV-1 genomic vector (pSG3 delta *env* backbone).

### Heat-Map Analysis Based on Clustering of Plasma Neutralization Activity Data

The plasma nAb responses at all time-points was determined by heat-map tool using hierarchical clustering, freely available at HIV-1 database (https://www.hiv.lanl.gov/content/sequence/HEATMAP/heatmap.html), that clustered the 28 baseline and 8 follow up HIV-1-infected samples and 11 HIV-1 viruses on the basis of natural log ID_50_ values.

### Statistical Analysis

Results are depicted as median and interquartile ranges. Spearman Rank test was used to evaluate the correlation between plasma levels of BLyS and geometric mean titers (GMTs) of neutralization in the infected children. *p*-values < 0.05 were considered significant. For comparison of test values between seronegative donors, LTNPs and progressors, Mann–Whitney test was used. For analysis of paired samples of HIV-1-infected children before and after initiation of ART, Wilcoxon signed rank test was applied. Analyses were performed using GraphPad PRISM 5.0 for Windows (GraphPad Software Inc.).

## Results

### Sociodemographic and Clinical Characteristics of HIV-1 Infected Children

A total of 38 ART naïve HIV-1 chronically infected children, and 25 healthy seronegative controls were recruited for the study. The HIV-1-infected children were documented to have acquired the infection through perinatal mode of transmission. All study groups were similar with respect to age, sex, race and modes of HIV-1 acquisition. Based on the years post infection and CD4 counts, they were categorized into ART-naïve 20 LTNPs and 18 progressors. Among the progressors, 8 were followed up 6–12 months after ART initiation. Mean age of progressors was lower than that of LTNPs (*p* < 0.0001). Progressors showed significantly lower CD4 counts (*p* = 0.001) and higher viral loads than that observed in LTNPs (*p* = 0.0002). The demographic profile of the HIV-1-infected children is summarized in Table 1.

**Table 1:** Demographic and clinical profile of the HIV-1-infected children.

Parameter	LTNPs (*n* = 20)	Progressors pre-ART (*n* = 18)	*p*-Value
Age (Y), median (range)	9.5 (6.9–14)	3 (1.2–7)	–
CD4 count, cells/μL, median (range)	780 (450–1964)	355 [age > 5 years] (103–356)	0.001
CD4 % (range)	–	20% [age < 5 years] (11–24%)	
Viral load, RNA copies/mL, median (range)	23,400 (4,080–2.6 × 10^5^)	585,680 (61835–3.15 × 10^7^)	0.0002

*n, number of subjects; Y, years; LTNPs, long-term non-progressors*.

### Lower Percentages of mDCs and pDCs and High Expression of BLyS on Surface of mDCs Observed in Progressors

**Figure 2:**
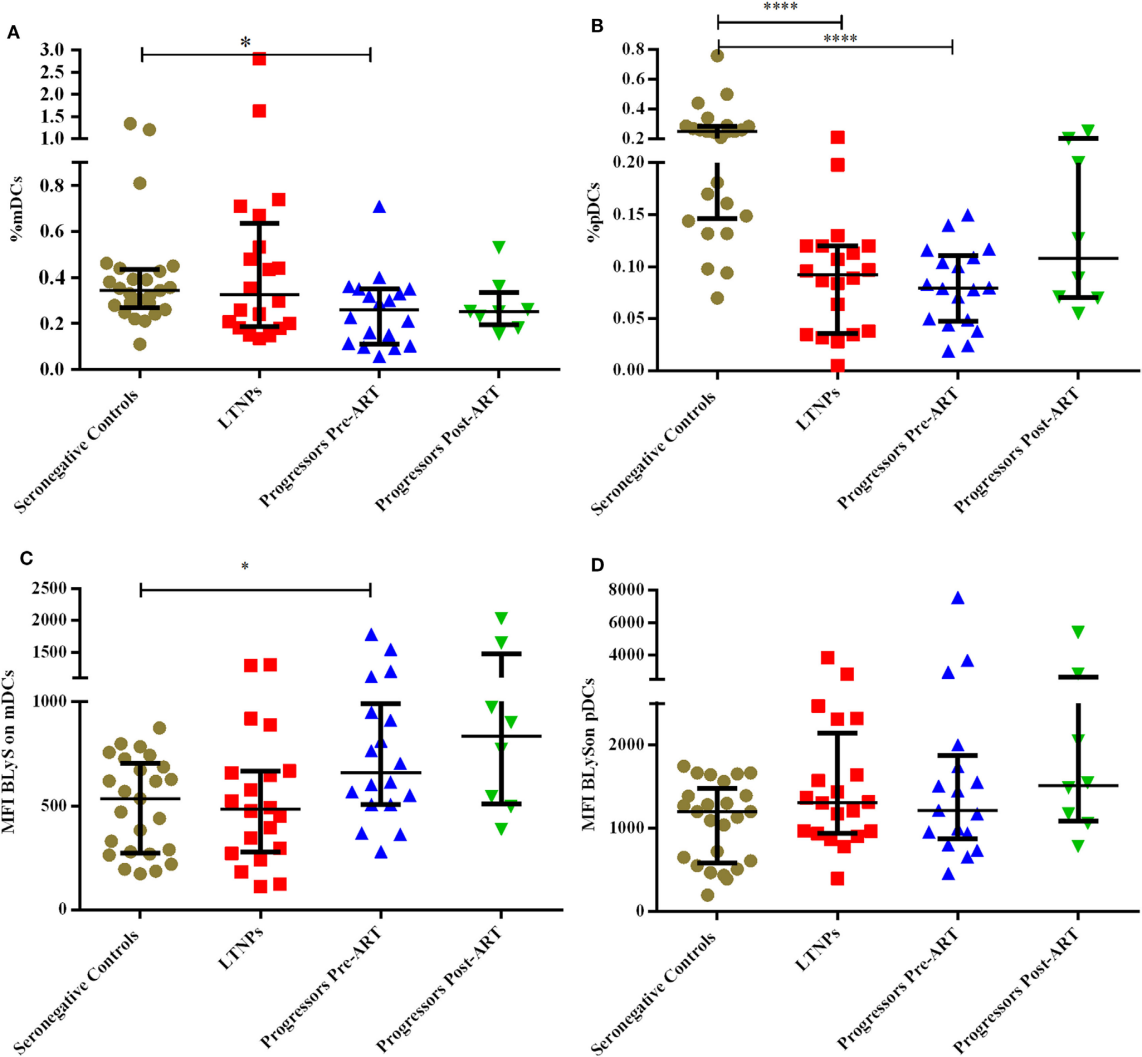
Monitoring of percentage of mDCs, pDCs, and B lymphocyte stimulator (BLyS) expression on surface of mDCs and pDCs in HIV-1 infected children at different stages of disease. **(A–D)** show comparison of % myeloid DCs (mDCs), % pDCs, MFI BLyS on mDCs, MFI BLyS on pDCs among 25 HIV-1 negative seronegative controls, 20 HIV-1-infected ART naive long-term non-progressors (LTNPs), 18 progressors pre-ART and 8 progressors post 6–12 months of ART, respectively. The *p-*values are calculated using Mann–Whitney *U* test for seronegative controls, LTNPs, progressors pre-ART. Wilcoxon signed rank test was used for paired analysis of progressors pre-ART and post 6–12 months of ART. The error bars show the median with the interquartile range. *p ≤ 0.05, ****p ≤ 0.0001.

The DCs gating experiments shown in Figure 1 are described in the legends. Progressors showed significantly lower % of mDCs as compared to seronegative controls (*p* = 0.015), with no significant differences between the LTNPs and seronegative controls (Figure 2A). Significantly lower % of pDCs was observed in progressors (*p* < 0.0001) and LTNPs (*p* < 0.0001) as compared to seronegative controls (Figure 2B). Surface expression of BLyS on mDCs was found to be significantly higher in progressors (*p* = 0.021) as compared seronegative controls (Figure 2C). Surface expression of BLyS on pDCs did not differ significantly among seronegative controls and HIV-1 infected LTNPs and Progressors (Figure 2D).

### Lower % of Naïve, Resting Memory (RM) B Cells and Higher % of Mature Activated (MA), Tissue-Like Memory (TLM) B Cells Observed in Progressors

**Figure 3:**
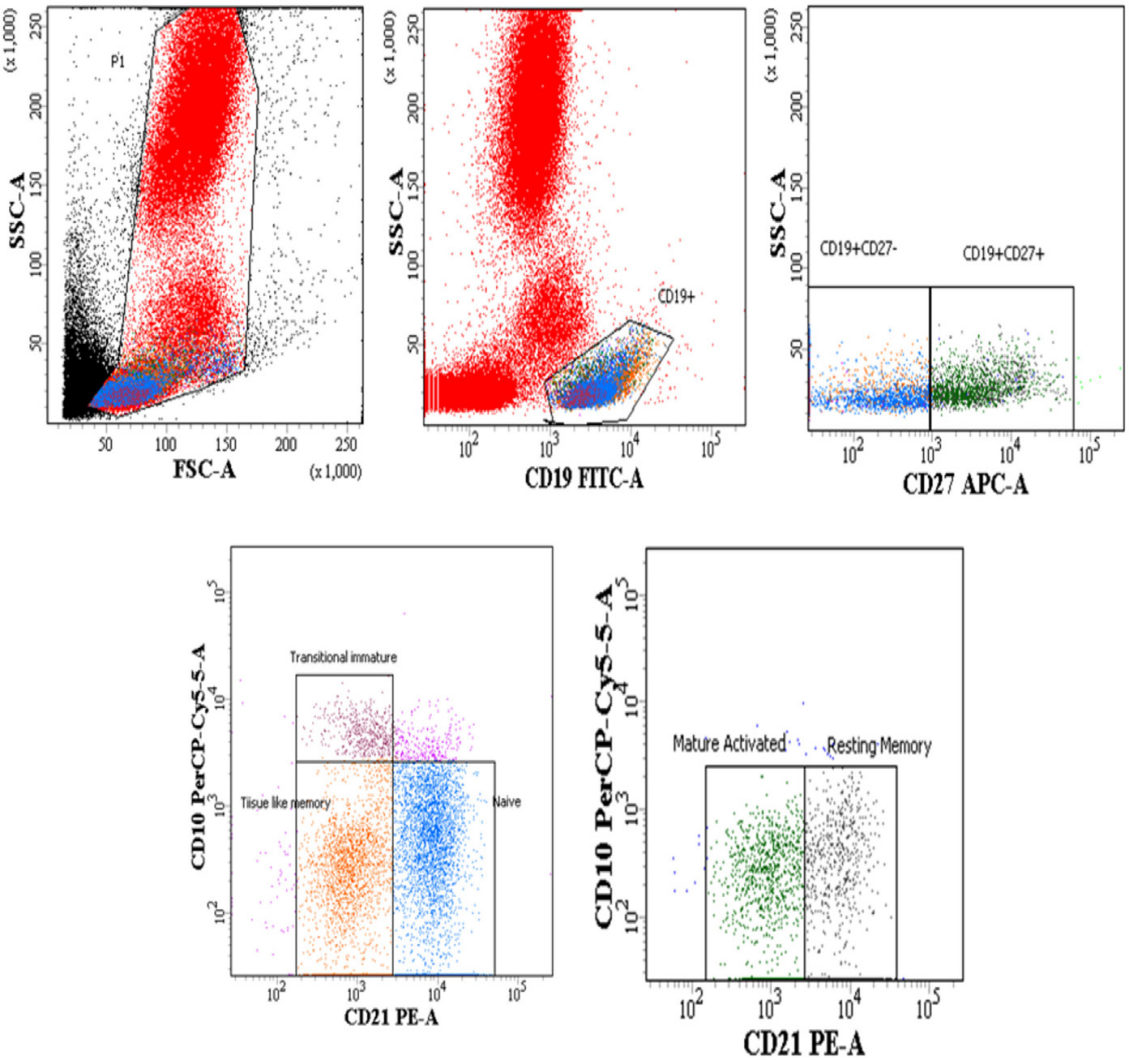
Flow-cytometric analysis of B cell subsets (gated on CD19^+^ B cells) in HIV-1-infected children at different stages of disease. Representative gating strategy for naïve B cells (CD19^+^ CD27^−^ CD10^−^ CD21^+^), tissue-like memory (TLM) B cells (CD19^+^ CD27^−^ CD10^−^ CD21^−^), transitional immature (TI) B cells (CD19^+^ CD27^−^ CD10^+^ CD21^−^) resting memory (RM) B cells (CD19^+^ CD27^+^ CD10^−^ CD21^+^) and mature activated (MA) B cells as (CD19^+^ CD27^+^ CD10^−^ CD21^−^). Lower left panel shows naïve, TI and TLM B cells gated on CD19^+^ CD27^−^ cells, lower right panel shows mature activated and resting memory B cells gated on CD19^+^ CD27^+^ cells. Percentages of B cell subsets are expressed as % of CD19^+^ B cells.

**Figure 4:**
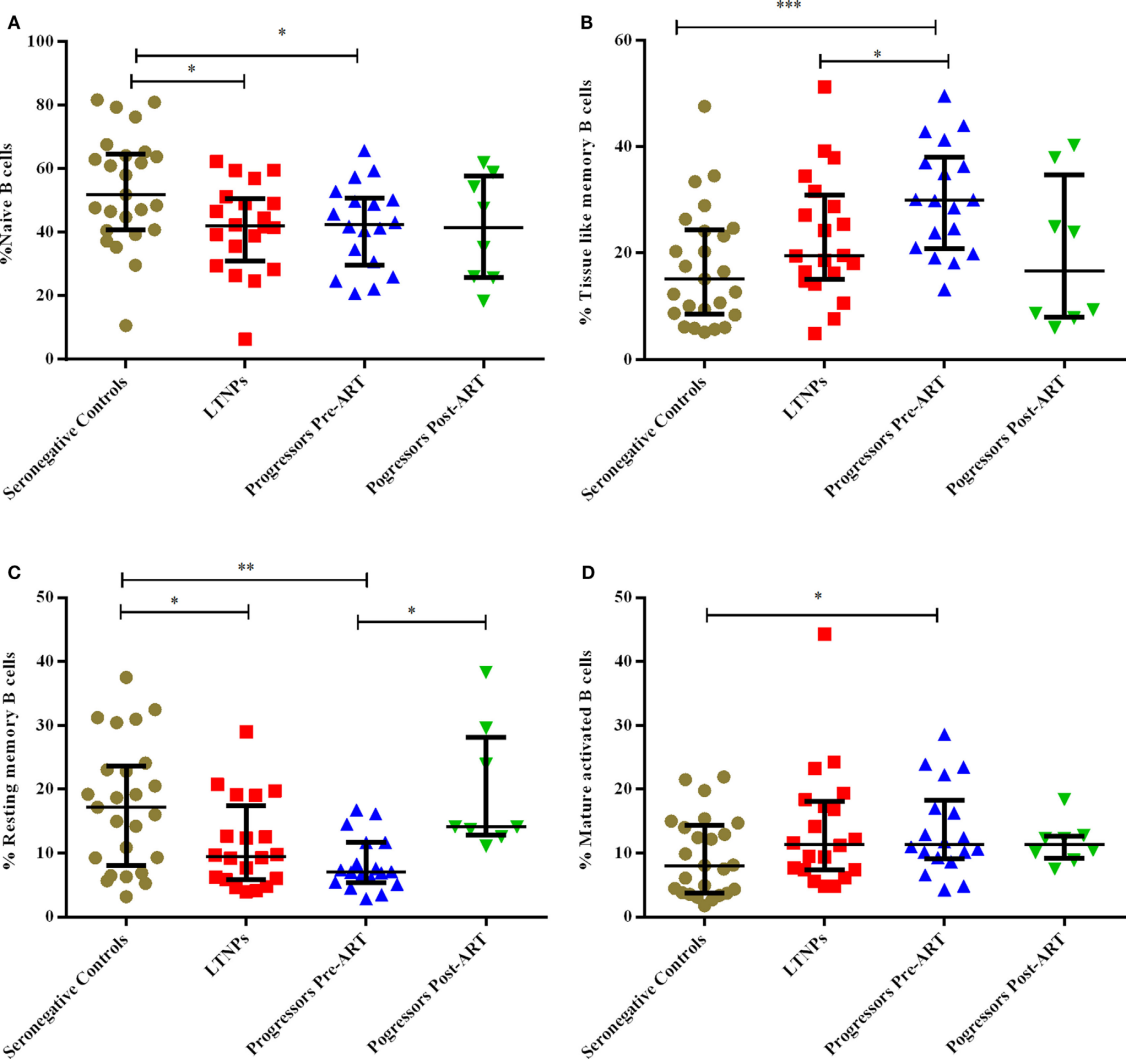
Monitoring of percentage of B cell subsets in HIV-1 infected children at different stages of disease. **(A–D)** show comparison of naïve B cells, tissue-like memory B cells, resting memory B cells, mature activated B cells among 25 HIV-1 negative seronegative controls, 20 HIV-1-infected ART naive long-term non-progressors (LTNPs), 18 progressors pre-ART and 8 progressors post 6–12 months of ART, respectively. The *p-*values are calculated using Mann–Whitney *U* test for seronegative controls, LTNPs, progressors pre-ART, Wilcoxon signed rank test was used for paired analysis of progressors pre-ART and post 6–12 months ART. The error bars show the median with the interquartile range. *p ≤ 0.05, **p ≤ 0.01, ***p ≤ 0.001.

The B cells gating experiments shown in Figure 3 are described in the legends. The percentages of naive B cells in all HIV-1-infected children recruited in this study were lower irrespective of disease stage than those observed in HIV-1 seronegative controls, reaching statistical significance in both LTNPs (*p* = 0.016) and progressors (*p* = 0.031) (Figure 4A). Further, % of TLM B cells (*p* = 0.0003) and mature activated (MA) B cells (*p* = 0.041), remained significantly higher in progressors compared to seronegative controls (Figures 4B,D). A significant decrease in resting memory (RM) B cell frequencies (Figure 4C) was observed in infected progressors as well as LTNPs (*p* = 0.032) compared to seronegative controls, with a more prominent decrease seen in progressors (*p* = 0.0015), indicating severe loss of resting memory B cells in progressors. Upon initiation of ART in eight progressors, resting memory B cells were significantly restored (*p* = 0.015) (Figure 4C; Figure S2A in Supplementary Material).

### Plasma Levels of B-Cell Growth Factors (BLyS, IL-4, IL-6, IL-10), IgA and IgG in HIV-1-Infected Children at Different Stages of Disease

**Figure 5:**
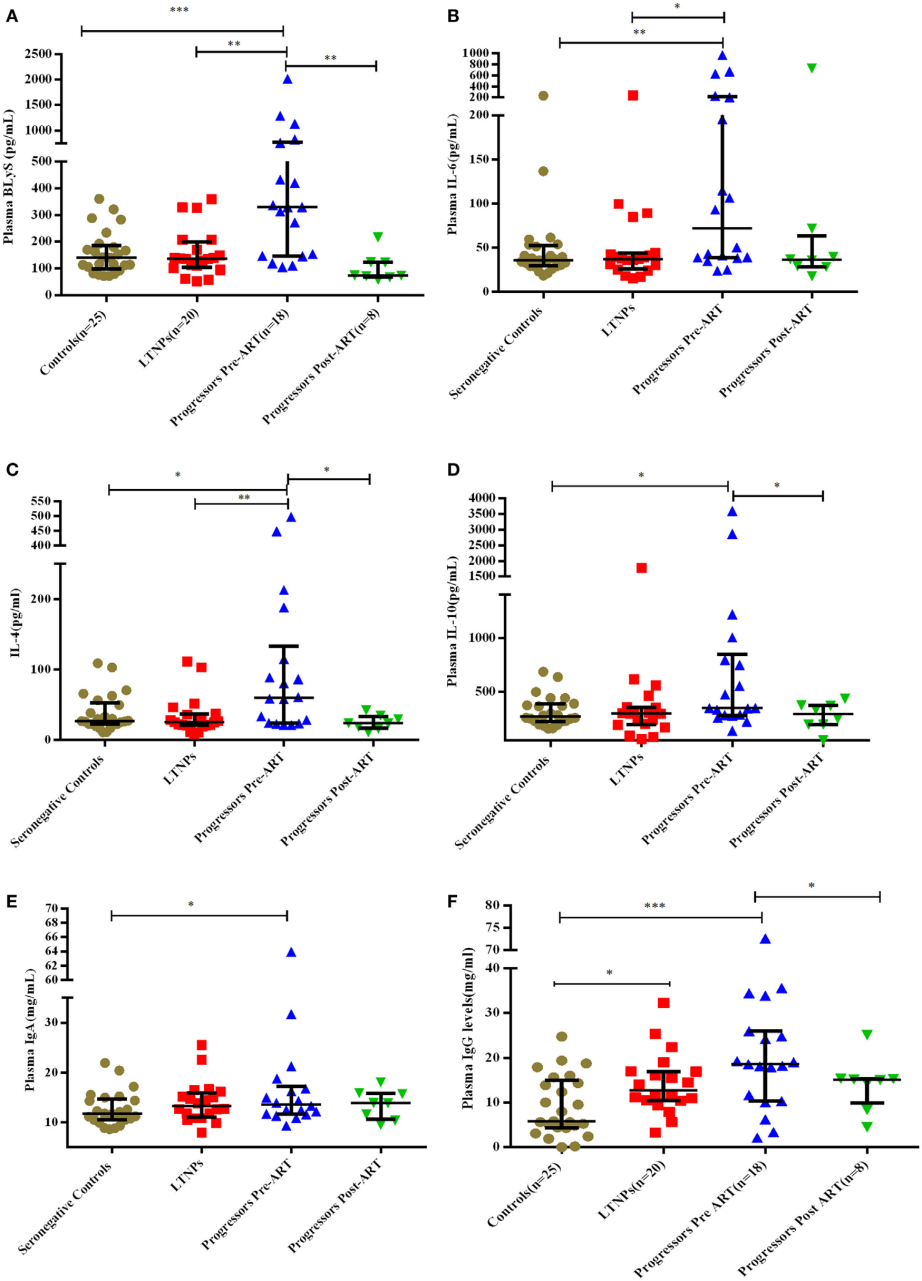
Plasma concentration of B-cell growth factors and immunoglobulins in HIV-1 infected children at different disease stages of disease. **(A–F)** show levels of B lymphocyte stimulator (BLyS), IL-6, IL-4, IL-10, IgA, and IgG among 25 HIV-1 seronegative controls, 20 HIV-1-infected ART naive long-term non-progressors (LTNPs), 18 progressors pre-ART and 8 progressors post 6–12 months of ART. The *p-*values are based on Mann–Whitney *U* test among seronegative donors, LTNPs, progressors pre-ART. Wilcoxon signed rank test was used for paired analysis of progressors pre-ART and post 6–12 months of ART. The error bars indicate median values with interquartile range. *p ≤ 0.05, **p ≤ 0.01, ***p ≤ 0.001.

Plasma levels of BLyS were higher in progressors compared to LTNPs and seronegative controls (*p* = 0.0007) (Figure 5A). Levels of BLyS decreased upon initiation of ART in progressors (*p* = 0.007) and were comparable with the levels in LTNPs (Figure 5A; Figure S2B in Supplementary Material). The plasma levels of IL-6 (*p* = 0.008), IL-4 (*p* = 0.019), IL-10 (*p* = 0.026), and IgA (*p* = 0.044) were higher in progressors than in seronegative controls (Figures 5B–E). The plasma IgG levels were high in progressors (0.0008) and in LTNPs (*p* = 0.014) as compared to seronegative controls (Figure 5F) while in progressors post 6–12 months of ART, decrease in levels of IL-4 (*p* = 0.039), IL-10 (*p* = 0.039), and IgG (*p* = 0.023) were observed (Figures 5C,D,F).

### Low ID_50_ Titers of Neutralizing Antibodies (nAbs) in Progressors Pre-ART vs. LTNPs and Significant Improvement in NAb Titers Post-ART

**Figure 6:**
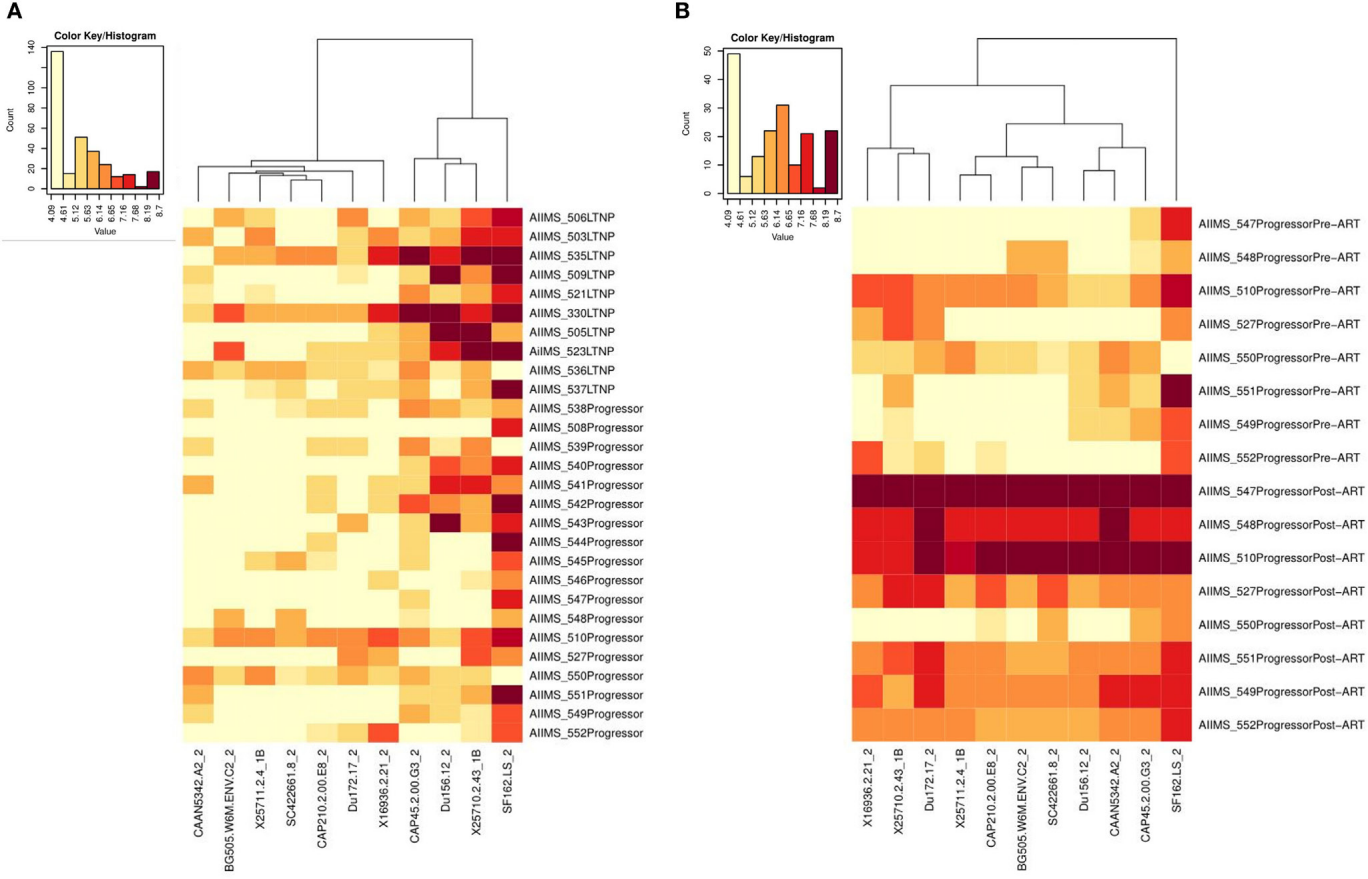
Heat-map analysis of ID_50_ titers of plasma samples from HIV-1-infected children based on hierarchical clustering and transformed natural log data of ID_50_ values. **(A)** shows comparison of ID_50_ titers of 10 ART naive long-term non-progressors (LTNPs) and 18 progressors, and **(B)** shows comparison of ID_50_ titers of 8 progressors pre-ART and post 6–12 months of ART against a panel of 11 virus strains. In the heat-map, each row shows the natural log ID_50_ values for each plasma sample, while the columns display the viruses. Darker color represents better neutralizing activity. Alpha numerals given after the underscore along with the viruses indicate the tier of the virus.

We compared the viral neutralization activity of plasma antibodies against a panel of 11 tier 1 and tier 2, clade A, B, and C viruses from 10 ART naïve LTNPs and 18 progressors pre-ART and follow-up samples of 8 progressors post 6–12 months of ART. Higher neutralization titers were observed in LTNPs as compared to progressors (darker the color, greater the ID_50_ values) (Figure 6A; Figure S3 in Supplementary Material). Progressors did not elicit effective Ag-specific NAb responses. A substantial improvement in viral neutralizing activity was observed on initiation of ART in eight progressors (Figure 6B; Figure S3 in Supplementary Material).

### Progressors Depicted Lower GMTs Compared to LTNPs against a Panel of 11 Pseudoviruses

**Figure 7:**
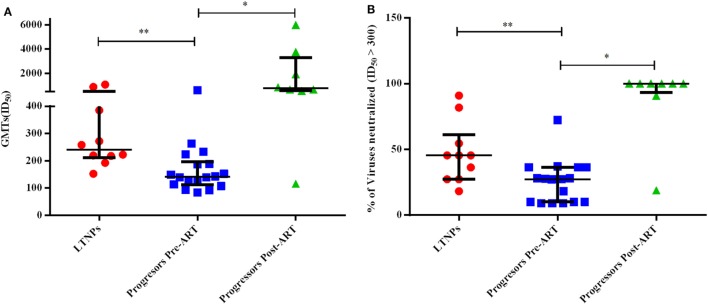
Evaluation of neutralization potency and % of viruses neutralized. **(A)** shows the comparison of geometric mean titers (GMTs), and **(B)** shows the comparison of % of viruses neutralized with ID_50_ ≥ 300 achieved amongst 10 ART naïve long-term non-progressors (LTNPs), 18 progressors pre-ART, and 8 progressors post 6–12 months of ART against a panel of 11 pseudoviruses. *p-*Values are based on Mann–Whitney *U* test among seronegative donors, LTNPs, progressors pre-ART, Wilcoxon signed rank test was used for paired analysis of progressors pre-ART and post 6–12 months ART. The error bars show the median with the interquartile range. *p ≤ 0.05, **p ≤ 0.01.

We compared GMTs using ID_50_ values of the plasma neutralizing activity of 10 ART naïve LTNPs and 18 progressors pre-ART and follow-up samples of 8 progressors post 6–12 months of ART. The LTNPs exhibited significantly higher GMTs of neutralizing activity compared to progressors (*p* = 0.001) against 11 tier 1 and 2 viruses of different clades (Figure 7A). To calculate % of viruses neutralized, ID_50_ > 300 was taken as cut off value. Progressors exhibited lower % of viruses neutralized vs. LTNPs (*p* = 0.009) (Figure 7B) and there was a significant improvement in GMTs (*p* = 0.015) (Figure 7A; Figure S2C in Supplementary Material) and % of viruses neutralized (*p* = 0.015) (Figure 7B; Figure S2D in Supplementary Material) in progressors, upon ART initiation.

### Higher Levels of BLyS Correlated with Poor Viral Neutralization

**Figure 8:**
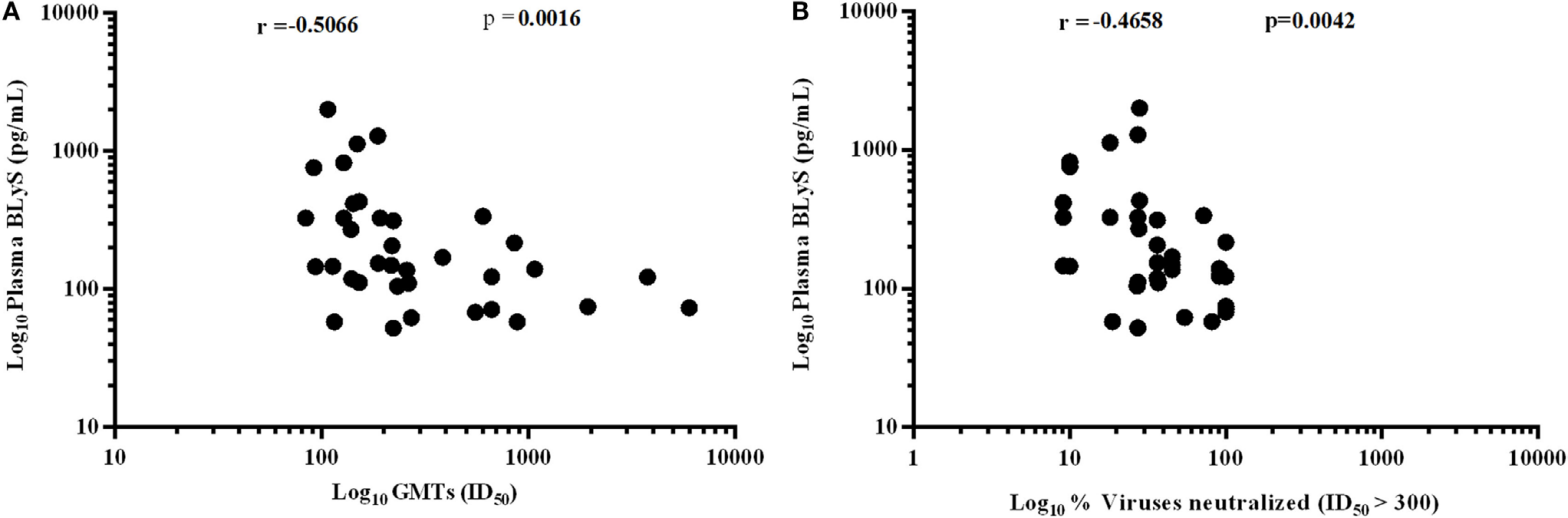
Association of HIV-1-specific neutralization response with B lymphocyte stimulator (BLyS). **(A)** shows correlation of plasma levels of BLyS with geometric mean titers (GMTs), and **(B)** shows correlation of plasma BLyS with % of viruses neutralized with ID_50_ ≥ 300 against a panel of 11 pesudoviruses of different clades and tiers. Correlation coefficient (r) was calculated using spearman rank correlation, for graphical representation *x*-axis, *y*-axis was converted to log scales.

A significant negative correlation was observed between plasma levels of BLyS vs. GMTs (*p* = 0.0016) (Figure 8A) and % viruses neutralized (*p* = 0.0042) (Figure 8B). High levels of BLyS in HIV-1 infected children may plausibly be one of contributory factors for poor viral neutralizing activity and faster disease progression in untreated progressors as compared to LTNPs with appropriate/basal levels of BLyS.

## Discussion

Antiretroviral therapy has to a great extent reduced mother-to-child transmission, however, many children continue to become infected vertically; especially in resource limited settings. In 2016, 160,000 children acquired HIV-1 infection, majority being from low- and middle-income countries (42). Furthermore, only 43% of eligible children received HAART in 2016. If left untreated, most HIV-1-infected children die within 2 years of age, probably owing to their inability to mount adequate immune responses. Elucidating the underlying mechanisms of immunological deficits in HIV-1-infected children will help in designing effective interventional strategies. Earlier studies have reported defects in DCs leading to alterations in B cell subsets in HIV-1-infected individuals (30, 35, 43, 44). An inverse correlation between BLyS (BAFF) released by mDCs and B cell numbers has been shown during primary HIV-1 infection (45). However, it is less clear how such alterations in DCs compromise B cell function in HIV-1 infection and disease progression.

B cell-associated cytokine CXCL13, has recently been proposed as a predictor for the development of cross nAbs in hyperacute HIV-1 C-infected women (29). The broadly neutralizing antibodies evolve after a minimum of 2–3 years of HIV-1 infection, only in a small subset of infected individuals (46, 47). It is important to delineate the factors promoting the development and persistence of such bnAbs in select individuals. Herein, we have the advantage of having a pediatric cohort comprising of antiretroviral naïve LTNPs with controlled disease and progressors, of which, a subset have been followed up post 6–12 months of ART. To the best of our knowledge, this is the first study conducted in subtype C HIV-1 chronically infected children, to show a link between altered expression of BLyS and antigen specific humoral immune response. The reduction in circulating DCs, resting memory B cells, and increase in MA and TLM B cells-associated with higher expression of BLyS in progressors of our study, is in accordance with that reported (15, 17, 24, 30, 35, 43, 44). Recently, it has been shown in mice that BlyS excess is associated with expansion of immature B cells (48, 49). However, in our cohort, transitional immature B cells were observed only in six seronegative controls, two LTNPs, and five progressors (Table S3 in Supplementary Material), and the influence of high BLyS on this B cell population could not be inferred. Disease progression in HIV-1 infection involves a complex set of interactions between viral and host factors leading to a chronic immune activation state. Inflammation has been shown to be a contributing factor, in addition to T cell activation, towards disease progression and mortality (13). In viremic individuals, it has long been recognized that viral replication may not solely be responsible for disease progression; immune activation status being an important determinant too (3). High plasma levels of BLyS, IL-4, IL-6, and IL-10 observed in the progressors in this study along with B-cell dysregulation plausibly favor the overall inflammatory burden and impair viral neutralization efficiency. The expression and secretion of BLyS have been shown *in vitro* to be potentiated by inflammatory cytokines, such as IL-2, IL-10, TNF-α, and IFN-γ (50, 51). Association of higher expression of BLyS on mDCs and plasma levels with lower % of memory B cells and poor viral neutralizing activity in progressors suggests that high BLyS influences the survival, tissue distribution, and differentiation of B cells, thereby affecting the ultimate production of Ag-specific bnAbs. Among the other cytokines studied, only a weak negative correlation was found between plasma IL-4 levels and GMTs of neutralization in the infected children (Figure S4 in Supplementary Material). Sriram et al. showed that *in vitro* stimulation of murine bone marrow-derived conventional dendritic cells (cDCs) by TLR7/9 ligands in presence of IL-4, mediates suppression of antiviral responses (IFNβ and IFN-responsive genes), resulting in increased permissiveness of cDCs to viral infection (42). Similar observations herein of progressors with high viral load and high IL-4 levels correlating with poor viral neutralizing activity suggests the plausible involvement of IL-4 in antigenic persistence leading to polyclonal B cell activation and poor viral neutralizing activity.

Reduction in BLyS levels in the progressors post 6–12 months of ART and its correlation with increase in memory B cells and improvement in neutralizing activity, indicates that optimal levels of BLyS may be one of the determinants for maintain B cell functionality. Moreover, the high GMTs of neutralization in progressors post 6–12 months ART, reach levels similar/higher than that found in the LTNPs in this study and in a previous study (52–54). The influence of varying levels of BLyS and viral neutralizing efficiency (GMT) of nAbs needs to be further evaluated in a larger pre- and post-ART cohort of infected children. The limit number of follow ups herein is a drawback of our study. Rouers et al. (37) have observed that ECs naturally preserve their memory B cell compartments and maintain HIV-1 specific memory B cell responses with a broader cross neutralizing capacity. Further assessment of BLyS levels in such ECs would provide valuable information. Hypergammaglobulinemia was observed in infected children at different disease stages, with significantly higher plasma IgG levels in progressors than LTNPs in this study. Earlier reports suggest that IgGs in HIV-1 infected individuals are polyclonal in nature and there is loss of antigen-specific humoral immunity, as has also been observed by us (55).

Restoration of memory B cell responses in progressors post ART in this study is in agreement with previous studies documenting the beneficial effect of early initiation of ART (23, 44, 56–58). In 2013, WHO conditionally recommended that all 2- to 5-year-old HIV-1-infected children be placed on HAART, based on studies that demonstrated improvement in clinical and virological parameters post ART (52). The present study furthers the beneficial effect of ART in early control of viremia in HIV-1-infected children and emphasizes the need to implement the new guidelines rigorously. Further, a detailed analysis of mDC subsets (CD1c^+^, CD141^+^) and monocytes contributing to HIV-1-specific B cell subsets (59), and their function will provide significant insight in this direction.

To summarize, a compromised B cell compartment and high levels of BLyS correlated with poor viral neutralizing Ab response in HIV-1-infected pediatric progressors. Our findings suggest that modulation of BLyS expression may be considered for therapeutic interventional strategies to prevent B cell dysfunction and systemic immune activation, that are the hallmarks of HIV-1 disease progression.

## Ethics Statement

The study was approved by the “Institute Ethics Committee” All India Institute of Medical Sciences (AIIMS), New Delhi (IEC/NP-269/2012 & RP-39/2012). All the experiments were carried out in accordance with relevant institutional ethics committee guidelines and regulations. Written informed signed consent forms were obtained from the parents/guardians of all the study subjects.

## Author Contributions

KL conceived and designed the study, edited and finalized the manuscript. RL, SKK, BD, and RS provided the HIV-1-infected pediatric samples. MS and KS provided the seronegative control samples. HA performed the research, data analyses, and wrote the manuscript. OC helped HA in flow-cytometry experiments. LK, SK, MM, and HA performed neutralization assays and contributed to manuscript editing. NM performed some of the ELISA experiments.

## Conflict of Interest Statement

The authors declare that the research was conducted in the absence of any commercial or financial relationships that could be construed as a potential conflict of interest.
