# Supplementary material for: Alterations in B Cell Compartment Correlate with Poor Neutralization Response and Disease Progression in HIV-1 Infected Children
Source: Front Immunol. 2017 Dec 1;8:1697. doi: 10.3389/fimmu.2017.01697 (PMC5717014; doi:10.3389/fimmu.2017.01697)
Supplement: Supplementary file 1 [file Data_Sheet_1.PDF]

Supplementary Material

Alterations in B cell compartment correlate with poor neutralization response and disease progression in HIV-1 infected children

Heena Aggarwal[1], Lubina Khan[1], Omkar Chaudhary[1], Sanjeev Kumar[1], Muzamil Ashraf Makhdoomi[1], Kanika Sharma[3], Ravinder Singh[2], Nitesh Mishra[1], Rakesh Lodha[2], Maddur Srinivas[3], Bimal Kumar Das[4], Sushil Kumar Kabra[2], Kalpana Luthra[1]*

*Correspondence: **Prof. Kalpana Luthra**: E-mail: kalpanaluthra@gmail.com

Supplementary Figures and Tables

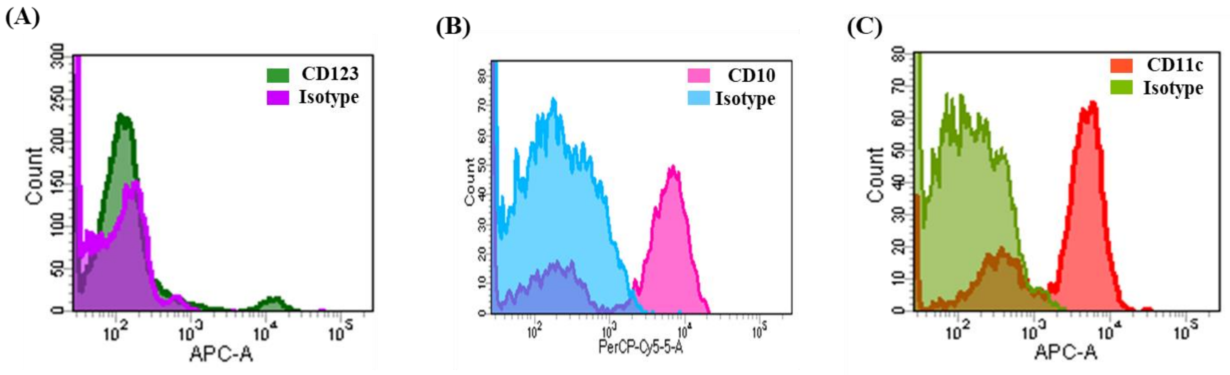

Figure S1. Isotype controls for antihuman CD123, CD10and CD11c antibodies. (A) Shows overlap of isotype control (IgG_1, κ- APC) for anti-human CD123 antibody and positive control for anti-human CD123 (APC) antibody. (B) Shows overlap of isotype control for anti-human CD10 (IgG_1, κ-PerCP Cy5.5) and positive control for antihuman CD10 (PerCP Cy5.5) antibody. (C) Shows overlap of isotype control for anti-human CD11c (IgG_1, κ-APC) and positive control for antihuman CD11c (APC) antibody.

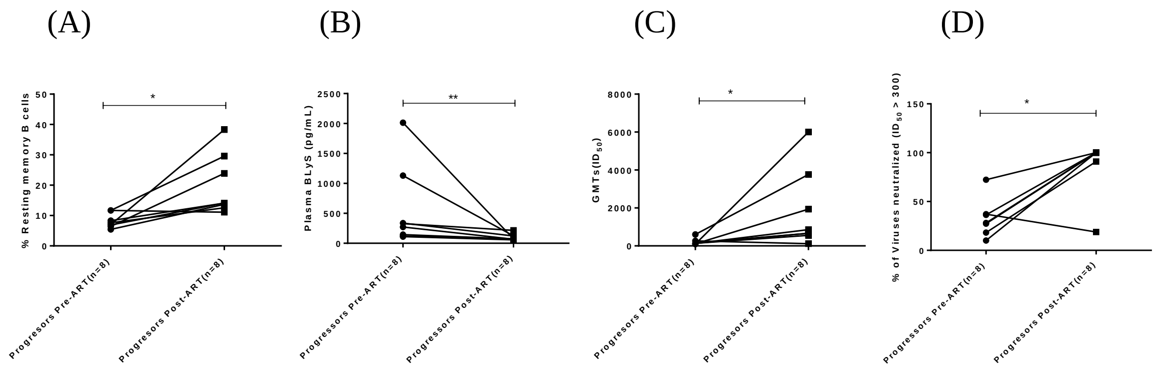

Figure S2. Paired analysis of various parameters in 8 progressors pre and post 6-12 months of ART. Panel (A) shows % of memory B cells, (B) shows plasma levels of BLyS, (C) shows GMTs of neutralization, (D) shows % of viruses neutralized.

	SUBTYPE C TIER-1		SUBTYPE B TIER-1	SUBTYPE C TIER-2					SUBTYPE B TIER-2		SUBTYPE A TIER-2
SAMPLE ID	25710-2.43	25711-2.4	SF162.LS	CAP210.2.00.E8	Du156.12	Du172.17	CAP45.2.00.G3	16936-2.21	CAAN5342.A2	SC422661.8	BG505.W6M.ENV.C2
**LTNPs**											
AIIMS_506	1083	259	3573	<60	261	531	328	98	<60	<60	351
AIIMS_503	1517	504	1567	<60	403	184	181	485	352	<60	<60
AIIMS_535	5093	309	4880	488	1925	275	5227	1767	79.2	488	357
AIIMS_509	528	<60	4267	<60	>6000	144	245	<60	176	<60	<60
AIIMS_521	395	109	1600	<60	256	<60	472	74.7	125	<60	<60
AIIMS_330	1825	309	>6000	445	4453	389	>6000	1450	245	307	1292
AIIMS_505	>6000	<60	421	<60	3893	75.8	392	248	<60	<60	<60
AiIMS_523	>6000	<60	>6000	171	1517	171	384	261	<60	<60	802
AIIMS_536	301	312	<60	248	136	237	515	109	307	312	213
AIIMS_537	344	133	>6000	<60	96	195	285	224	<60	192	<60
**PROGRESSORS**											
AIIMS_538	261	<60	443	264	397	261	496	<60	184	160	<60
AIIMS_508	<60	<60	1742	<60	<60	68.3	<60	72	<60	<60	<60
AIIMS_539	611	<60	84.2	248	152.8	176	531	<60	208	<60	<60
AIIMS_540	720	<60	1975	<60	875	<60	219	80	<60	<60	<60
AIIMS_541	1927	<60	548	168	1775	69	224	251	424	<60	<60
AIIMS_542	424	<60	4160	213	533	61	788	219	93.3	95	<60
AIIMS_543	307	<60	1458	<60	4640	299	269	80	<60	<60	<60
AIIMS_544	<60	<60	>6000	173	<60	77.3	173	<60	<60	<60	<60
AIIMS_545	<60	259	1167	120	<60	74	251	<60	<60	293	<60
AIIMS_546	136	<60	501	<60	<60	<60	98.7	173	72.5	<60	<60
**PRE-ART PROGRESSORS**											
AIIMS_547 Pre-ART	62	<60	1408	<60	<60	<60	256	77.5	<60	<60	<60
AIIMS_548 PreART	<60	<60	381	<60	<60	67.5	155	<60	<60	360	328
AIIMS_510 PreART	1142	469	3147	515	240	523	533	1058	256	464	531
AIIMS_527 PreART	975	<60	499	<60	<60	528	98	336	<60	<60	<60
AIIMS_550 PreART	245	629	<60	256	224	336	336	251	710	165	243
AIIMS_551 PreART	296	<60	4800	<60	203	<60	208	<60	288	<60	<60
AIIMS_549 PreART	120	<60	1158	<60	203	<60	416	62	269	<60	<60
AIIMS_552 PreART	122	<60	1183	125	<60	253	<60	1183	<60	89	<60
**POST-ART PROGRESSORS**											
AIIMS_547 PostART	>6000	>6000	>6000	>6000	>6000	>6000	>6000	>6000	>6000	>6000	>6000
AIIMS_548 PostART	1533	1342	1858	1408	2117	>6000	1742	1450	3893	1408	1525
AIIMS_510 PostART	1958	3333	4160	4373	>6000	>6000	3600	1567	5413	3787	4320
AIIMS_527 PostART	1667	413	564	925	429	1850	563	477	477	925	333
AIIMS_550 PostART	64	<60	477	131	<60	84	389	80.8	<60	363	<60
AIIMS_551 PostART	958	483	1650	620	581	1692	589	600	477	437	320
AIIMS_549 PostART	432	656	1400	653	499	1317	1750	1233	1892	487	541
AIIMS_552 PostART	627	523	1325	421	451	549	597	557	659	373	421

<60	61-200	201-500	501-1000	1001-6000	>6000

Figure S3. ID_{50} titers of plasma neutralization potential against 11 HIV-1 Env-pseudoviruses.

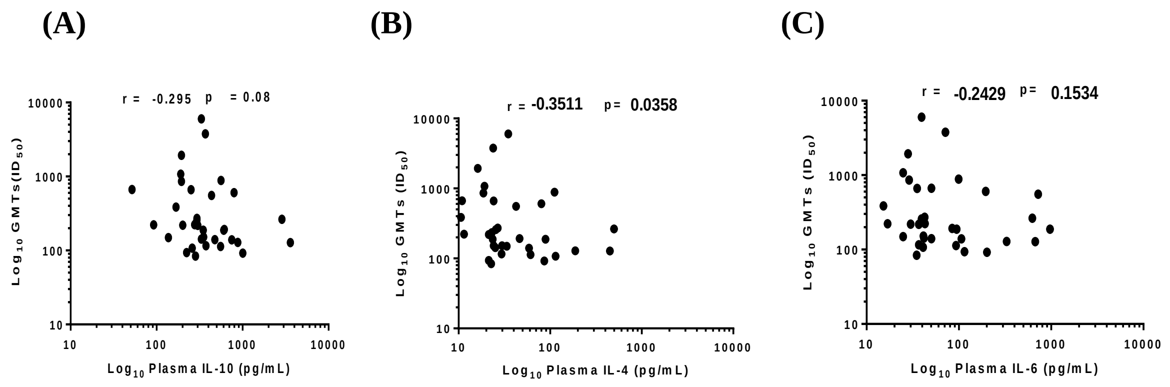

Figure S4. Association of HIV-1 specific neutralization response with various cytokines. The panel shows correlation of plasma levels of (A) IL-10 with GMTs of neutralization, (B) IL-4 with GMTs of neutralization, (C) IL-6 with GMTs of neutralization.

Table S1. Panel of antibodies used for enumeration of %mDCs, %pDCs and % B cell subsets.

Myeloid DCs

Antigen	Clone	Fluorescent Dye	Manufacturer
Lineage 1 (cocktail of CD3, CD19, CD20, CD14, CD16, CD56)	CD3, clone SK7<br>CD16, clone 3G8<br>CD19, clone SJ25C1<br>CD20, clone L27<br>CD14, clone MφP9<br>CD56, clone NCAM16.2	FITC	BD Biosciences
HLA-DR	G46-6	PerCPCy5.5	BD Biosciences
CD11c	3.9	APC	Biolegend
CD257 (mBAFF/BLyS)	T7-241	PE	Biolegend

Plasmacytoid DCs

Antigen	Clone	Fluorescent Dye	Manufacturer
Lineage 1 (cocktail of CD3, CD19, CD20, CD14, CD16, CD56)	CD3, SK7<br>CD16, 3G8<br>CD19, SJ25C1<br>CD20, L27<br>CD14, MφP9<br>CD56, NCAM16.2	FITC	BD Biosciences
HLA-DR	G46-6	PerCPCy5.5	BD Biosciences
CD123	6H6	APC	Biolegend
CD257 (mBAFF/BLyS)	T7-241	PE	Biolegend

B cells

Antigen	Clone	Fluorescent Dye	Manufacturer
CD19	HIB19	FITC	BD Biosciences
CD21	B-ly4	PE	BD Biosciences
CD27	M-T271	APC	BD Biosciences
CD10	HI10a	PerCPCy5.5	BD Biosciences

Table S2. Panel of HIV-1 Env pseudovirus used in neutralization assays.

#	Virus ID	Subtype	Tier	Country of origin
1	Du172.17	2	C	South Africa
2	Du156.12	2	C	South Africa
3	25711-2.4	1B	C	India
4	25710-2.43	1B	C	India
5	CAP45.2.00.G3	2	C	South Africa
6	CAP210.2.00.E8	2	C	South Africa
7	16936-2.21	2	C	India
8	SC422661.8	2	B	Trinidad and Tobago
9	CAAN5342.A2	2	B	USA
10	SF162.LS	1A	B	USA
11	BG505.W6M.ENV.C2	2	A	Kenya

Table S3. Percentages of transitional immature B cells.

Seronegative Donor	% immature B cells
1	5.09
2	5.8
3	9.03
4	5.47
5	7.19
6	15.2
LTNPs	
1	5.01
2	7.31
Progressors	
1	6.3
2	10.02
3	11.5
4	13.9
5	6.62
